# Physical activity and breast cancer risk in Chinese women

**DOI:** 10.1038/bjc.2011.370

**Published:** 2011-09-20

**Authors:** A Pronk, B-T Ji, X-O Shu, W-H Chow, S Xue, G Yang, H-L Li, N Rothman, Y-T Gao, W Zheng, C E Matthews

**Affiliations:** 1 Division of Cancer Epidemiology and Genetics, National Cancer Institute, 6120 Executive Blvd, EPS 8100, MSC 7240, Bethesda, MD 20892, USA; 2 Department of Medicine, Vanderbilt Epidemiology Center, Institute for Medicine and Public Health, Vanderbilt-Ingram Cancer Center, Vanderbilt University School of Medicine, Nashville, TN 37203, USA; 3 Department of Epidemiology, Shanghai Cancer Institute, Shanghai, People's Republic of China

**Keywords:** breast cancer, physical activity, exercise, occupational, critical period

## Abstract

**Background:**

The influence of different types and intensities of physical activity on risk for breast cancer is unclear.

**Methods:**

In a prospective cohort of 73 049 Chinese women (40–70 years), who had worked outside the home, we studied breast cancer risk in relation to specific types of self-reported and work history-related physical activity, including adolescent and adult exercise and household activity and walking and cycling for transportation. Occupational sitting time and physical activity energy expenditure were assigned based on lifetime occupational histories.

**Results:**

In all, 717 incident breast cancer cases were diagnosed. Breast cancer risk was lower for women in the lowest quartile of average occupational sitting time and in the highest quartile of average occupational energy expenditure (adjusted hazard ratio (HR): 0.81 and 0.73, respectively, *P*⩽0.05). Adult exercise at or above the recommended level (8 metabolic equivalent (MET) h per week per year) was associated with lower risk (adjusted HR: 0.73, *P*<0.05) in post-menopausal women. Analysis of joint effects showed that having both an active job and exercise participation did not confer an additional benefit. Other common daily activities were not associated with lower risk.

**Interpretation:**

These findings suggest that both exercise and occupational activity are associated with lower breast cancer risk, which supports current health promotion campaigns promoting exercise.

The association between physical activity and breast cancer has been investigated by numerous observational studies. Breast cancer risk reductions of 25–30% and 15–20% in the most active women compared with the least active women were reported by two recent reviews, which included 62 (Friedenreich and Cust, 2008) and 48 (Monninkhof *et al*, 2007) relevant epidemiological studies, respectively. The abundant epidemiological evidence has been classified as ‘convincing’ by the International Agency for Research on Cancer (IARC, 2002). However, there are still a number of questions of public health relevance related to the association between physical activity and breast cancer.

First, the majority of the first-generation studies between physical activity and breast cancer focussed on leisure time physical activity (exercise) and did not always consider the influence of other types of physical activity. Second, the current research focus has shifted towards breast cancer risk reduction in population subgroups and critical time periods in life during which the impact of physical activity may vary. Findings to date are mostly inconclusive (Friedenreich and Cust, 2008). And finally, the role of physical activity at work has been primarily studied in industrialised countries. Less is known about the impact of physical activity in economically less developed countries, where occupational activities may comprise a relatively larger proportion of energy expenditure in daily life (Monda *et al*, 2008).

To comprehensively assess the role of physical activity, including occupational and non-occupational activities, in breast cancer risk, we examined data from a large cohort of Chinese women, of whom over 99% had held a job outside the home. We sought to evaluate the associations with specific types of physical activity, and to explore whether breast cancer risk varies by age periods of exposure.

## Materials and methods

### Study population

The Shanghai Women's Health Study (SWHS) is a population-based prospective cohort study conducted in seven urban communities in Shanghai, China (Zheng *et al*, 2005). Between 1996 and 2000, 74 942 women (40–70 years) were recruited with a participation rate of 92.7%. The cohort was followed for occurrence of cancer and other chronic diseases by a combination of biennial in-person interviews and annual record linkage to the Shanghai Cancer Registry and Shanghai Vital Statistics database. The response rates for the first (2000–2002), second (2002–2004), and third (2004–2007) follow-up surveys were 99.8%, 98.7%, and 96.7%, respectively. Medical charts from the diagnostic hospital were reviewed to verify the diagnosis. For the present analyses, women who had a history of cancer at study entry (*n*=1576), never held a job outside the home (*n*=274), or did not have a complete work history (*n*=43) were excluded. The study was approved by the Institutional Review Boards of the participating institutes in China and the United States.

### Data collection

In-person interviews were conducted at baseline by trained interviewers using a structured questionnaire to elicit information on exercise and other forms of non-occupational physical activity, lifetime occupational history, demographic background, socioeconomic status, family history of cancer, and reproductive factors. Menopausal status was defined as stopping of menstruation for at least 12 months, including natural and surgically induced menopause. Menopausal status was updated at each follow-up survey, and the updated menopausal status was used in the analyses.

### Non-occupational physical activity assessment

Information about non-occupational physical activity was assessed by interview. Exercise during adolescence (13–19 years) was assessed as the number of years of participation and the weekly duration. Summary estimates of exercise duration in adolescence (h per week per year) were calculated. In adulthood, up to three exercise activities were reported for the 5-year period before the interview, and for each activity reported, the weekly duration and years of participation were obtained. Time spent in household activities (h per day) was assessed and information about active transportation (h per day) was gathered via specific questions regarding walking and cycling to and from work, and walking and cycling to do daily errands. Non-occupational activities were evaluated for the year before the interview. Summary estimates of exercise energy expenditure in adulthood (metabolic equivalent (MET)-h per week per year) were calculated by multiplying standard MET values associated with each exercise reported (Ainsworth *et al*, 2000) with exercise duration (h per week per year), and then creating a sum for all exercise activities. Similarly, summary estimates for non-exercise activities in adulthood were calculated using the following MET values based on relevant activities in the Compendium (Ainsworth *et al*, 2000): household activities (2 METs), walking for transportation (3.3 METs), and cycling for transportation (4 METs), and 0.075 MET h per flight of stairs, and that have been used in previous studies (Matthews *et al*, 2003, 2007). One MET hour is roughly equivalent to 15 min of participation in a moderate intensity (4 METs) activity (Ainsworth *et al*, 1993). As noted above, we have evaluated the reliability and validity of the SWHS physical activity questionnaire and found that it was generally valid; however, reproducibility of non-exercise physical activities tended to be lower than for exercise participation (Matthews *et al*, 2003).

### Occupational physical activity assessment

Occupational histories included the name of the work place, job title including main duties and products, and year started and year ended for each job held longer than 1 year. These were self-completed and later reviewed for completeness and accuracy by trained interviewers during in-person interviews. Each occupation was coded (three digits) according to the Chinese National Standard Occupational and Industry Codes Manual (1986). Occupational physical activity was assessed by using a job exposure matrix, which assigned occupation codes into categories of low, medium, and high sitting time and low, medium, and high energy expenditure. Criteria for classification of the total of 300 specific occupation codes in the study were used in earlier surveys and have been modified by industrial hygienists based on the work environments in China (Vetter *et al*, 1992; Chow *et al*, 1993; Zheng *et al*, 1993). Categories of sitting time were defined as jobs with low (<20% of working hours), medium (20–80%), and high (>80%) sitting time. Categories for energy expenditure were defined as low (<8 kJ per min, e.g., sitting with only hand work, moderate one arm work, and light two arm work), medium (8–12 kJ per min, e.g., walking on flat surface with speed of 3 km per h, heavy one arm work, or moderate two arm work), and high (>12 kJ per min, e.g., walking on flat surface with speed of >4 km per h, heavy two arm work or light to heavy body work) energy expenditure. For each job in a subject's work history, the median level of sitting time (1, 4, and 7 h per day for low, moderate, and high, respectively) and energy expenditure (4, 10, and 16 kJ per min for low, moderate, and high, respectively) was multiplied by the number of years worked in that particular job. These products were summed over each person's work history to calculate the lifetime cumulative sitting time (h per day per year) and lifetime cumulative energy expenditure (kJ per min per year). Average annual sitting time (h per day) and energy expenditure (kJ per min) summary measures were calculated by dividing the lifetime cumulative measure by the total years of employment. Lifetime number of years in jobs with high physical activity was computed by summing the total number of years in jobs categorised as low sitting time (<2 h per day) or high energy expenditure (>12 kJ per min).

### Statistical analyses

The association for risk of breast cancer was evaluated using Cox proportional hazards regression (proc PHREG), with age as the time scale and stratification by birth cohort (5-year intervals) using SAS Version 9.1. (SAS Institute, Cary, NC, USA). Hazard ratios (HRs) and 95% confidence intervals (95% CIs) were calculated by physical activity quantiles (quartile or tertile distributions in the cohort). For exercise activity also the current recommended level of 8 MET day per week per year by the Physical Activity Guidelines Advisory Committee (2008) (US Department of Health and Human Services) was used a cut-point. Analyses were performed for all women and for the population stratified on menopausal status (at the last follow-up) and body mass index (BMI; above and below the median of 23.7). To test for linear trends in HRs across groups, we created score variables representing the median of each category and included them in the models as continuous variables. We tested the primary activity exposures for possible violations of the proportional hazards assumption, and the assumption was satisfied for these exposures.

Effective time periods for occupational physical activity were investigated by separately modelling average energy expenditure and sitting time defined by tertile distributions during several age periods (<25, 25–34, 35–44, and 45+ years) as categorical variables. Subjects who did not work during the age period under study were excluded from the analyses in the particular age group.

Unless stated otherwise, HRs were adjusted for education level, family history of breast cancer, age at first birth (<23, 23–25, 26–27, and 28+), and number of pregnancies (0 or 1, 2, 3, 4+). The potential effects of other covariates were also assessed, including menopausal status, age at menarche (<14, 14, 15, and 16+), and total caloric intake (<1412, 1412–1643, 1644–1908, and 1909+ kcal per day), and BMI (kg m^−2^). Additional adjustment for these factors did not affect the risk estimates meaningfully and were therefore not included in the final models. In addition, to study the independent effect of the physical activity exposure of primary interest, the other activity exposure variables were consecutively added into the models. For example, when exercise in adulthood was the primary exposure, we also adjusted for potential effects of the non-exercise activities and average occupational activity. Similarly, in models estimating the effect of occupational activity, we adjusted for all non-occupational activity. Results from these analyses did not suggest that controlling for the additional effects of other types of activity affected the risk estimated for the exposure of primary interest. Thus, we report results only for the models including the primary exposure of interest in the final models.

Since physical activity energy expenditure is a modifiable component of the energy balance equation and has been associated with reduced weight gain, we considered BMI to be an important element in causal pathways between physical activity and breast cancer (McTiernan, 2008), and therefore, present the results without controlling for this variable.

## Results

Of 73 049 women followed for an average of 9.0 years, 717 were newly diagnosed with an invasive malignant neoplasm of the breast (ICD-9 code 174). Compared with the cohort, a greater proportion of cases reported a high school or higher education, a history of breast cancer in first-degree relatives, and later age at first live birth (Table 1).

Breast cancer risk tended to be inversely associated with the highest level of adult non-occupational physical activities (Table 2). The associations with the exception of adult exercise, however, were not statistically significant and were reduced to near null after adjustment for potential confounding factors, particularly educational attainment. Adolescent exercise was also not associated with breast cancer risk after adjusting for confounding factors (data not shown).

**Figure 1:**
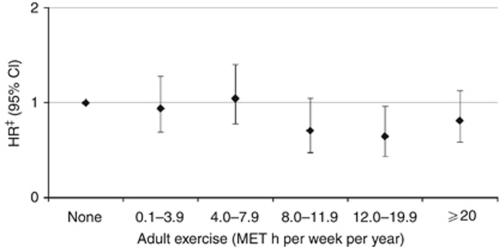
Association: hazard ratio (HR) and 95% confidence interval (95% CI) between adult exercise (up to three exercise activities over 5 years preceding the interview) energy expenditure and incident breast cancer in 54 289 post-menopausal women of the Shanghai Women's Health Study.

After stratification for menopausal status, a significant reduction in breast cancer risk associated with adult exercise was mainly confined in post-menopausal women. Compared with women who exercised little, post-menopausal women who exercised at or above the current recommended level of 8 MET h per week per year had a 30% lower risk (HR=0.73, 95% CI=0.57, 0.92) of breast cancer compared with women reporting lower levels of exercise (<8 MET h per week per year). A corresponding reduction in risk for this level of exercise was not observed among pre-menopausal women (HR=1.25, 95% CI=0.77, 2.01). Post-menopausal women who exercised at levels that were more than twice the recommended minimum levels did not appear to have any additional reduction in risk (Figure 1). Stratification on BMI showed a decreased risk of breast cancer associated with adult exercise at or above the currently recommended levels in women with a BMI of 23.73 kg m^−2^ or greater (HR=0.66, 95% CI=0.49, 0.88), but not with a BMI below this level (HR=1.02, 95% CI=0.75–1.40). For walking, cycling, and household chores and adolescent exercise, stratification by menopausal status or BMI did not result in any consistent differences in the strength or direction of the associations by BMI for the exposures examined.

On average, women were employed for 29 years in 2.3 jobs. Jobs with high, medium, and low energy expenditure comprised 7%, 52%, and 41% of the total of 2 096 414 years worked, respectively. In contrast, 39%, 42%, and 19% were spent in jobs with low, medium, and high sitting time. Increasing lifetime occupational sitting time and decreasing occupational energy expenditure were significantly associated with breast cancer risk when adjusting for age only (Table 3). The average, cumulative, and duration indices for sitting time and energy expenditure showed similar significant trends of decreasing risk with increasing physical activity. The associations for sitting time were attenuated after further adjusting for other potential confounding factors, but the trends remained significant for average sitting time, duration in jobs with low sitting time, and duration in jobs with high energy expenditure. Similarly to non-occupational physical activity, the attenuation in risk after adjustment for potential confounders could be attributed mainly to educational attainment. No clear differences were observed between pre- and post-menopausal women or women with high and low BMI (data not shown).

We also examined the joint effect of occupational sitting time by reported exercise participation in adulthood in post-menopausal women (Table 4). Compared with women who were both occupationally inactive and had inadequate exercise, breast cancer risk was ∼30% lower among women who had either active jobs or adequate exercise, but having both an active job and adequate exercise did not confer further reduction in risk. No statistically significant interaction was observed.

Investigation of the effect of average occupational physical activity during four age periods is shown in Table 5. Breast cancer risk was non-significantly lower in women in the highest tertile of physical activity for all age groups, without clear differences between the age groups.

## Discussion

In our large prospective cohort study of Chinese women who were employed outside the home, we found a lower risk of breast cancer among those who held more active jobs and those who exercised above the current recommended level of 8 MET h per week per year. The inverse association with exercise was confined mainly in post-menopausal women. Furthermore, analysis of joint effects with occupational sitting time suggests that the reduction in risk with exercise was most apparent among post-menopausal women who held sedentary jobs. We found no association with other common daily activities, such as housework and walking and cycling for transportation.

Recent Physical Activity Guidelines for Americans (Committee, 2008) recommended that adults accumulate ∼8–17 MET h per week of moderate-vigorous physical activity to prevent chronic disease. We examined the dose–response for adult exercise and observed a reduction in risk within the recommended range in post-menopausal women only. Exercise at levels beyond the recommended range (i.e., >8 MET h per week per year) did not appear to confer additional benefit in our population. This was also observed by the Women's Health Initiative Observational study, a cohort of almost 70 000 post-menopausal women aged 50–79 years (McTiernan *et al*, 2003). Interestingly, the exercise effect appeared to be confined to post-menopausal women with higher BMI levels. This finding is in contrast to earlier studies which have found effects mostly in leaner women from the United States and Europe (Thune *et al*, 1997; McTiernan *et al*, 2003). It may be that the relatively low BMI levels in this population (i.e., median 23.73 kg m^−2^) partially accounts for the present finding, although we have reported an inverse association for exercise among women with lower and higher BMI levels in a previous case–control study from Shanghai (Matthews *et al*, 2001a). Notably, we did not find evidence for effect modification by BMI on our occupational physical activity exposures in the present study. Our null results for exercise among pre-menopausal women are consistent with studies indicating a weak or little association among younger women (Friedenreich and Cust, 2008). We do not believe that this null finding is due completely to a lack of statistical power since 212 of our cases were pre-menopausal and the HRs remained close to 1.0 in each exposure level.

Our results of a borderline significant decrease in breast cancer risk for women with high levels of occupational physical activity are consistent in the direction and magnitude of the association with several other prospective studies that have reported a 15–25% lower risk with occupational activity (Thune *et al*, 1997; Moradi *et al*, 1999; Dirx *et al*, 2001; Rintala *et al*, 2002, 2003; Mertens *et al*, 2006). The results of the joint effect analyses of occupational physical activity and adult exercise showed a lower risk with active jobs among women who did not exercise and suggested that exercise is particularly important for women who held sedentary jobs. However, having both an active job and exercise did not confer an additional reduction in risk. We considered developing a measure of total activity in the present study, reflecting both occupational and non-occupational exposures, but the variation in the length of the time frame for the last job reported in relation to our non-occupational exposures made logical combinations of the two exposures difficult. Future studies with measurements for occupational and non-occupational physical activity that can be integrated more effectively needed to better evaluate the joint effects of these two important exposures on risk for breast cancer.

In addition to occupational activity and exercise, non-exercise activities such as housework and walking and cycling for transportation are also recommended as a means of increasing overall activity levels. In contrast to studies that have reported inverse associations between breast cancer and higher levels of household activities (Friedenreich *et al*, 2001; Tehard *et al*, 2006; Lahmann *et al*, 2007) or walking (Suzuki *et al*, 2008), we did not find evidence of an inverse association in this population. At least two other prospective studies (Patel *et al*, 2003; Tehard *et al*, 2006) also did not observe this relation. The null findings may have resulted from measurement error in our assessment of non-exercise activity (Ferrari *et al*, 2007). While our evaluation of the physical activity questionnaire was generally supportive of its validity, reproducibility of non-exercise physical activities tended to be lower than for exercise participation, suggesting they may have been more difficult to recall, or that they may have been more variable over time (Matthews *et al*, 2003). In addition, the assessment of our non-exercise physical activities only reflected the past year of exposure, while our assessment of exercise and occupational physical activity reflected multiple years of exposure, which may provide a better estimate of long-term exposure for occupational activity. Finally, it is possible that the variability in household and transportation activity was low in this population.

To our knowledge, our study is the first population-based prospective cohort study that collected lifetime occupational histories. This has resulted in a more comprehensive measure of long-term occupational exposure than in previous cohort studies, which have confined their exposure assessment to the current or main job or to a specific time period. In addition, questionnaire-based estimates were available for several sources of non-occupational physical activity, allowing for more thorough assessment of the role of physical activity. Other advantages include the high response rate, the availability of detailed information on potential confounding variables and a large proportion of women who had held a job (over 99%), and long (median 26 years) work histories. In addition, most previous studies have focussed on European and North American populations. Our study also suggests an inverse effect in a population that differs in several important respects from these populations: Asian race, lower BMI, few cohort members report a family history of breast cancer, and breast cancer incidence is low. There are also some limitations to our study. Information on non-occupational physical activity was self-reported and limited to the past year (for daily activities) or past 5 years (exercise). We only gathered information about three or fewer exercise activities, which could result in an underestimation of exercise. However, only 1.6% of the women reported three or more exercise activities. This information may be susceptible to systematic reporting errors (Adams *et al*, 2005) and misclassification due to the natural variability of behaviours over time (Wareham *et al*, 2000; Matthews *et al*, 2001b). In addition, a limitation to using job codes for the assessment of occupational physical activity is that a single activity level is linked to the specific job title, and we cannot account for changes in activity levels within a specific job class. Therefore, we may have underestimated the activity levels for job types that have experienced reductions in activity within the job class. The most likely effect of this kind of exposure misclassification (assuming the inverse relationship between activity and breast cancer is true) would be an underestimation of the actual effect. The classification system should reflect a great deal of the modernisation that has taken place in China in recent decades. In addition, there is also a potential for selection bias as a result of women with functional limitations to avoid physically demanding jobs resulting in exposure misclassification.

It has been suggested that there might be critical time periods during which physical activity may be particularly important, since physical activity has been hypothesised to affect breast cancer risk through changes in menstrual characteristics, body size, serum hormone levels, or immune function (Gammon *et al*, 1998). Two previous occupational studies did not show obvious differences for timing of exposure (D’Avanzo *et al*, 1996; Levi *et al*, 1999), while two others suggested that only occupational physical activity during women's reproductive years was associated with decreased breast cancer (Moradi *et al*, 2000; Peplonska *et al*, 2008). The lack of an effect by critical time periods in our study may be explained by the fact that the time span over which women in our cohort had worked (roughly age 18–50 years) did not or only partially cover adolescence and the perimenopause for most women. In addition, because of the relatively small number of jobs held by the women in this study (median 2) there may not be sufficient variation in occupational physical activity within study subjects for a meaningful evaluation of the effect of time period.

In conclusion, this population-based prospective study suggests that women who held active jobs were at lower risk of breast cancer in a population in which breast cancer risk is generally lower than in western countries. In post-menopausal women, this risk may be modified by moderate intensity exercise at or above the recommended level (8 MET h per week per year). These findings provide support for current health promotion campaigns that encourage women to participate in moderate intensity exercise, which may be particularly important for women that work in sedentary work environments.

## Figures and Tables

**Table 1:** Baseline (1996–2000) characteristics of the Shanghai Women's Health Study cohort by breast cancer status

	**Cohort (*n*=73 049)**	**Case (*n*=717)**
Age at cohort entry (years)	52.5 (9.1)	52.7 (8.8)
Breast cancer in first-degree relative (%)	2	4
		
*Education (%)*		
Elementary or lower	21	13
Middle school	37	32
High school	28	35
College or higher	14	19
		
*Pre-menopausal (%)* a	26	30
Age at menarche (years)	14.9 (1.7)	14.8 (1.8)
Age at first life birth (years)	25.9 (4.1)	26.8 (4.2)
Number of pregnancies	2.8 (1.5)	2.6 (1.4)
Number of sisters	1.7 (1.4)	1.8 (1.4)
		
BMI (kg m^−2^)	24.0 (3.4)	24.2 (3.4)
Total caloric intake (kcal per day)	1686 (407)	1683 (375)

Abbreviation: BMI=body mass index.

Values are mean (standard deviation) or percent of total reporting (%).

a Menopausal status was updated during follow-up (last follow-up 2004–2006).

**Table 2:** Association between adulthood non-occupational activity (MET-h per week per year) and breast cancer risk in the Shanghai Women's Health Study: hazard ratio's (HR) and 95% confidence intervals (CIs)

			**Age adjusted**		**Fully adjusted** a	
	**Cohort (*N*=73** **049)**	**Cases (*N*=717)**	**HR**	**95% CI**	**HR**	**95% CI**
*Walking* b						
0 to <28.1	16 730	185	1.0		1.0	
28.1 to <46.2	13 741	142	0.95	0.76, 1.18	0.91	0.73, 1.14
46.2 to <69.3	20 761	191	0.85	0.69, 1.03	0.90	0.74, 1.11
69.3+	21 808	199	0.85	0.69, 1.03	0.95	0.77, 1.16
			*P*-trend=0.13		*P*-trend=0.85	
						
*Riding bike* b						
None	56 139	548	1.0		1.0	
>0 to <10.0	4040	44	1.16	0.85, 1.58	1.12	0.82, 1.53
10.0 to <14.0	4034	46	1.21	0.89, 1.65	1.16	0.85, 1.58
14.0 to <21.4	4590	42	0.97	0.71, 1.34	0.93	0.67, 1.28
21.4+	4244	37	0.93	0.67, 1.31	0.89	0.63, 1.25
			*P*-trend=0.03		*P*-trend=0.56	
*Household chores* b						
0 to <28.0	13 518	151	1.0		1.0	
28.0 to <42.0	23 637	230	0.86	0.70, 1.06	0.88	0.72, 1.08
42.0+	35 893	336	0.82	0.68, 0.99	0.89	0.73, 1.09
			*P*-trend=0.06		*P*-trend=0.34	
*Adult exercise activities* c						
None	47 429	471	1.0			
>0 to <4.6	6482	73	1.12	0.87, 1.43	1.05	0.82, 1.35
4.6 to <9.2	6436	69	1.04	0.81, 1.35	1.02	0.79, 1.32
9.2 to <17.6	6396	45	0.68	0.50, 0.93	0.69	0.50, 0.94
17.6+	6306	59	0.90	0.68, 1.19	0.92	0.69, 1.21
			*P*-trend=0.03		*P*-trend=0.04	
						
*All non-occupational physical activity*						
0 to <74.3	18 148	191	1.0		1.0	
74.3 to <100.2	18 246	195	1.01	0.83, 1.24	1.07	0.87, 1.30
100.2 to <131.5	18 334	167	0.87	0.71, 1.07	0.96	0.78, 1.18
131.5+	18 321	164	0.86	0.70, 1.06	0.98	0.79, 1.21
			*P*-trend=0.08		*P*-trend=0.64	

Abbreviation: MET=metabolic equivalent.

a Adjusted for age, education, family history of breast cancer, age at first birth, and number of pregnancies.

b In the year preceding the interview.

c Up to three exercise activities over 5 years preceding the interview.

**Table 3:** Association between occupational physical activity and breast cancer risk in the Shanghai Women's Health Study: hazard ratio's (HR) and 95% confidence intervals (CIs)

			**Age adjusted**		**Fully adjusted** a	
	**Cohort (*N*=73 049)**	**Cases (*N*=717)**	**HR**	**95% CI**	**HR**	**95% CI**
*Average sitting time (h per day)*						
⩾4.00	34 855	420	1.0		1.0	
⩾3.69 to <4.00	1679	17	0.86	0.53, 1.40	0.92	0.57, 1.50
⩾1.20 to <3.69	18 287	150	0.69	0.57, 0.84	0.82	0.67, 1.00
<1.20	18 228	130	0.61	0.50, 0.74	0.81	0.65, 1.01
			*P*-trend=<0.0001		*P*-trend=0.04	
						
*Cumulative sitting time score (h per day per year)*						
⩾137	18 265	223	1.0		1.0	
⩾94 to <137	18 348	214	0.97	0.80, 1.17	1.07	0.88, 1.30
⩾36 to <94	18 718	156	0.70	0.57, 0.86	0.91	0.72, 1.14
<36	17 718	124	0.60	0.48, 0.74	0.84	0.65, 1.08
			*P*-trend=<0.0001		*P*-trend=0.15	
						
*Duration in jobs with <2 h per day sitting time (years)* b						
0	28 528	353	1.0		1.0	
>0 to <11	14 873	145	0.80	0.66, 0.98	0.87	0.71, 1.06
⩾11 to <25	16 529	129	0.66	0.54, 0.82	0.83	0.67, 1.04
⩾25	13 119	90	0.56	0.44, 0.70	0.74	0.58, 0.96
			*P*-trend=<0.0001		*P*-trend=0.02	
						
*Average energy expenditure (kJ per min)*						
<4.64	18 227	232	1.0		1.0	
⩾4.64 to <9.61	18 250	202	0.88	0.73, 1.07	1.00	0.82, 1.22
⩾9.61 to <10.00	27 031	221	0.66	0.55, 0.80	0.95	0.76, 1.18
⩾10.00	9541	62	0.53	0.40, 0.70	0.73	0.53, 0.99
			*P*-trend=<0.0001		*P*-trend=0.14	
						
*Cumulative energy expenditure (kJ per min per year)*						
<144	17 315	204	1.0		1.0	
⩾144 to <228	19 103	222	0.99	0.82, 1.20	1.02	0.84, 1.23
⩾228 to <300	18 365	147	0.69	0.56, 0.86	0.84	0.67, 1.05
⩾300	18 266	144	0.66	0.53, 0.82	0.87	0.68, 1.09
			*P*-trend=<0.0001		*P*-trend=0.09	
						
*Duration in jobs with high energy expenditure >12 kJ per min (years)* b						
0	57 830	593	1.0		1.0	
>0 to <6	4755	42	0.88	0.65, 1.22	0.87	0.63, 1.20
⩾6 to <10	5025	51	0.98	0.73, 1.32	0.96	0.71, 1.30
⩾10	5439	31	0.56	0.39, 0.81	0.66	0.46, 0.96
			*P*-trend=0.004		*P*-trend=0.04	

a Adjusted for age, education, family history of breast cancer, age at first birth, and number of pregnancies.

b Duration is divided into tertiles and compared with no time in such positions.

**Table 4:** Joint effects: hazard ratio (HR) and 95% confidence interval (95% CI) of adult exercise (below and at or above the recommended level of 8 MET h per week per year) and occupational sitting time (tertiles) in 54 289 post-menopausal women of the Shanghai Women's Health Study

	**Adult exercise below recommended level of 8 MET h per week per year**				**Adult exercise above recommended level of 8 MET h per week per year**			
**Occupational sitting time**	**Cohort**	**Cases**	**HR^a^**	**95% CI**	**Cohort**	**Cases**	**HR** a	**95% CI**
⩾4 h per day	20 367	261	1.0		6115	54	0.65	0.48, 0.87
2.2–3.9 h per day	7844	49	0.61	0.45, 0.84	1857	12	0.60	0.33, 1.07
⩽2.1 h per day	13 363	99	0.82	0.63, 1.07	4743	30	0.68	0.45, 1.03

a Adjusted for age, education, family history of breast cancer, age at first birth, and number of pregnancies.

**Table 5:** Association between occupational physical activity in certain age periods and breast cancer risk in the Shanghai Women's Health Study: adjusted^a^ hazard ratio's (HR) and 95% confidence intervals (CI)

	**Sitting time average**			**Energy expenditure average**		
	**Cohort (cases)**	**HR**	**95% CI**	**Cohort (cases)**	**HR**	**95% CI**
*Before age 25 years*						
Group 1^b^	8402 (102)	1.0		18 436 (218)	1.0	
Group 2^b^	20 019 (211)	0.87	0.69, 1.11	29 563 (245)	0.92	0.75, 1.12
Group 3^b^	31 628 (253)	0.79	0.62, 1.01	12 050 (103)	0.84	0.64, 1.08
		*P-trend*=0.06			*P-trend*=0.17	
						
*Age 25–34 years*						
Group 1^b^	14 225 (160)	1.0		26 335 (328)	1.0	
Group 2^b^	29 976 (346)	1.04	0.86, 1.26	37 398 (320)	0.92	0.77, 1.10
Group 3^b^	26 768 (191)	0.8	0.7, 1.0	7236 (49)	0.73	0.53, 1.00
		*P-trend*=0.11			*P-trend*=0.10	
						
*Age 35–44 years*						
Group 1^b^	16 822 (191)	1.0		29 715 (359)	1.0	
Group 2^b^	31 687 (348)	0.99	0.83, 1.19	39 433 (336)	0.96	0.81, 1.14
Group 3^b^	23 884 (172)	0.85	0.68, 1.07	3245 (16)	0.63	0.38, 1.06
		*P-trend*=0.19			*P-trend*=0.22	
						
*After age 45 years*						
Group 1^b^	12 877 (158)	1.0		23 611 (280)	1.0	
Group 2^b^	26 967 (290)	0.95	0.78, 1.15	30 702 (273)	1.12	0.92, 1.36
Group 3^b^	16 579 (114)	0.81	0.62, 1.07	2110 (9)	0.64	0.32, 1.28
		*P-trend*=0.15			*P-trend*=0.72	

a Adjusted for age, education, family history of breast cancer, age at first birth, and number of pregnancies.

b Groups based on tertiles of average exposure in the total population: sitting time: >4, >2 to ⩽4, 0 to ⩽2 h per day; energy expenditure: 0 to ⩽6, 6 to ⩽10, >10 kJ per min.
